# Supplementary material for: Single-spin qubits in isotopically enriched silicon at low magnetic field
Source: Nat Commun. 2019 Dec 3;10:5500. doi: 10.1038/s41467-019-13416-7 (PMC6890755; doi:10.1038/s41467-019-13416-7)
Supplement: Supplementary file 1 — Supplementary Information [file 41467_2019_13416_MOESM1_ESM.pdf]

Supplementary Information : Single-spin qubits in isotopically enriched silicon at low magnetic field

R. Zhao,[1,*] T. Tanttu,[1] K. Y. Tan,[2] B. Hensen,[1] K. W. Chan,[1] J. C. C. Hwang,[1,†] R. C. C. Leon,[1] C. H. Yang,[1] W. Gilbert,[1] F. E. Hudson,[1] K. M. Itoh,[3] A. A. Kiselev,[4] T. D. Ladd,[4] A. Morello,[1] A. Laucht,[1] and A. S. Dzurak[1]

[1] *Centre for Quantum Computation & Communication Technology, School of Electrical Engineering & Telecommunications, University of New South Wales, Sydney, New South Wales 2052, Australia*

[2] *QCD Labs, QTF Centre of Excellence, Department of Applied Physics, Aalto University, 00076 AALTO, Finland*[‡]

[3] *School of Fundamental Science and Technology, Keio University, 3-14-1 Hiyoshi, Kohoku-ku, Yokohama 223-8522, Japan*

[4] *HRL Laboratories, LLC, 3011 Malibu Canyon Rd., Malibu, CA 90265, USA*

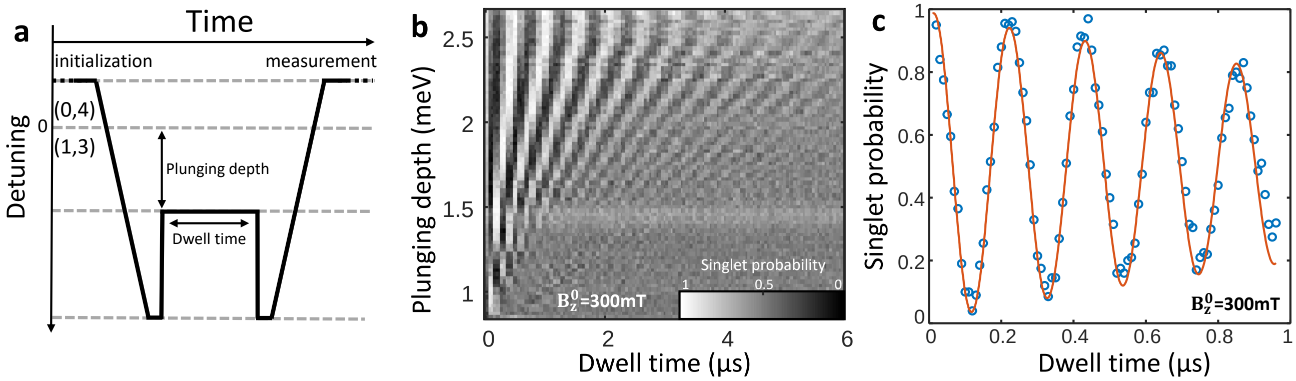

Supplementary Figure 1 | Exchange driven oscillations. **a**, Schematic diagram of the pulse sequence to measure the exchange oscillations. First, we initialize the double quantum dots (DQDs) in the $|\uparrow\downarrow\rangle$ state as described in Fig. 2a. Second,we enable the exchange coupling between the two dots by pulsing to the shallow detuning region. This will drive the coherent oscillations between the $|\uparrow\downarrow\rangle$ and $|\downarrow\uparrow\rangle$ states. We then stop the exchange driven oscillations by pulsing back to the deep detuning region. Finally, another adiabatic pulse brings the DQDs from (1,3) back to (0,4) region and completes the spin measurement, during which the $|\uparrow\downarrow\rangle$ state is converted to singlet and the $|\downarrow\uparrow\rangle$ state is converted to triplet. **b**, Singlet probability plotted as a function of plunging depth and dwell time at the shallow detuning region. **c**, A single trace of exchange driven oscillation with plunging depths of 1.9 meV. The red solid line indicates is a fit to the Rabi's formula with maximum visibility of 99.3±2.3 %.

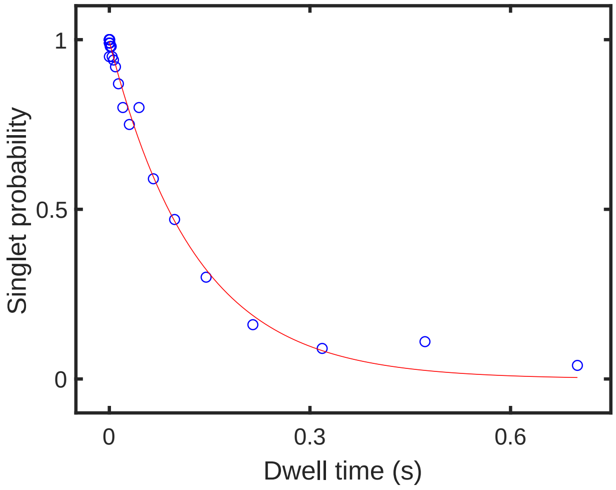

Supplementary Figure 2 | T_1 relaxation measurement and extraction of maximum visibities of $|\uparrow\downarrow\rangle$ and $|\downarrow\downarrow\rangle$ states. Decay of the $|\uparrow\downarrow\rangle$ state to the $|\downarrow\downarrow\rangle$ state as a function of dwell time. The red line is a fit to an exponential decay, indicating a relaxation time of 129±9 ms. Each data point is derived from 200 single-shot singlet-triplet readout events. All measurements are performed in an external dc magnetic field of 300 mT. We observe the mean of the singlet probability of these eight measurements to be 99.5±0.7%. This is the maximum experimentally observed visibility for the $|\uparrow\downarrow\rangle$ state and is consistent with the readout fidelity reported. Similarly, we extract the maximum readout visibility for the $|\downarrow\downarrow\rangle$ state to be 96.8±1.0% by setting the dwell time to 0.7 sec. This value could be limited by the 120 mK electron temperature of our system. We estimate the probability of the $|\downarrow\downarrow\rangle$ state to be thermally excited to the $|\uparrow\downarrow\rangle$ state to be $P_{\mathrm{excited}} = 1 - \exp(-g\mu_{\mathrm{B}}B_z^0/k_{\mathrm{B}}T_{\mathrm{electron}}) = 3.4\%$, where $g \approx 2$ is the gyromagnetic ratio of the electron, μ_{B} is the Bohr magneton, k_{B} is the Boltzmann constant and T_{electron} is the electron temperature of the device. Here, the error associated with the relaxation time stands for the 95% confidence interval derived from the uncertainties of data fitting.

* Present address: National Institute of Standards and Technology, 325 Broadway, Boulder, CO 80305, United States.
† Present address: Research and Prototype Foundry, The University of Sydney, Sydney, NSW 2006, Australia.
‡ Present address: IQM Finland Oy, Vaisalantie 6 C, 02130 Espoo, Finland
